# QUAliFiER: An automated pipeline for quality assessment of gated flow cytometry data

**DOI:** 10.1186/1471-2105-13-252

**Published:** 2012-09-28

**Authors:** Greg Finak, Wenxin Jiang, Jorge Pardo, Adam Asare, Raphael Gottardo

**Affiliations:** 1 Fred Hutchinson Cancer Research Center, 1100 Fairview Avenue North, Seattle, WA 98109, USA; 2 Tolerance Assays and Data Analysis, the Immune Tolerance Network, University of California, San Francisco, 3 Bethesda Metro Center, Bethesda, 20814, MD, USA; 3 Department of Statistics, University of Washington, Box 354322, Seattle, 98195, WA, USA

**Keywords:** Flow cytometry, Quality assessment, BioConductor package

## Abstract

**Background:**

Effective quality assessment is an important part of any high-throughput flow cytometry data analysis pipeline, especially when considering the complex designs of the typical flow experiments applied in clinical trials. Technical issues like instrument variation, problematic antibody staining, or reagent lot changes can lead to biases in the extracted cell subpopulation statistics. These biases can manifest themselves in non–obvious ways that can be difficult to detect without leveraging information about the study design or other experimental metadata. Consequently, a systematic and integrated approach to quality assessment of flow cytometry data is necessary to effectively identify technical errors that impact multiple samples over time. Gated cell populations and their statistics must be monitored within the context of the experimental run, assay, and the overall study.

**Results:**

We have developed two new packages, **flowWorkspace** and **QUAliFiER** to construct a pipeline for quality assessment of gated flow cytometry data. **flowWorkspace** makes manually gated data accessible to BioConductor’s computational flow tools by importing pre–processed and gated data from the widely used manual gating tool, **FlowJo** (Tree Star Inc, Ashland OR). The **QUAliFiER** package takes advantage of the manual gates to perform an extensive series of statistical quality assessment checks on the gated cell sub–populations while taking into account the structure of the data and the study design to monitor the consistency of population statistics across staining panels, subject, aliquots, channels, or other experimental variables. **QUAliFiER** implements SVG–based interactive visualization methods, allowing investigators to examine quality assessment results across different views of the data, and it has a flexible interface allowing users to tailor quality checks and outlier detection routines to suit their data analysis needs.

**Conclusion:**

We present a pipeline constructed from two new R packages for importing manually gated flow cytometry data and performing flexible and robust quality assessment checks. The pipeline addresses the increasing demand for tools capable of performing quality checks on large flow data sets generated in typical clinical trials. The **QUAliFiER** tool objectively, efficiently, and reproducibly identifies outlier samples in an automated manner by monitoring cell population statistics from gated or ungated flow data conditioned on experiment–level metadata.

## Background

Flow cytometry (FCM) is a high-throughput technology that offers rapid quantification of a set of physical and chemical characteristics for a large number of cells in a sample. The technology is widely used in health research and treatment, including for monitoring of infection, diagnosis of cancers like lymphoma and leukaemia, and auto–immune diseases [1-9]. It is also used for cross-matching organs for transplantation and in research involving stem cells, vaccine development, apoptosis, phagocytosis, and a wide range of cellular properties including phenotype, cytokine expression, and cell-cycle status [10-15]. Importantly, clinical trials in these fields often use flow cytometry to monitor the immune system or the progression of a disease over time, generating large amounts of data in the process.

Variation in instrumentation, antibody staining, reagent lots, and other technical problems can crop up over time and manifest themselves as biases in the extracted cell subpopulation statistics or fluorescence intensities. Such errors are neither obvious nor easy to detect via the examinations of dot plot outputs from individual FCS files that are performed as part of regular, daily quality control procedures in a flow cytometry core. Careful and systematic examination of gated populations over time and in the context of the larger study design together with follow–up analysis of experimental metadata is often necessary to identify the problematic samples as well as the underlying cause of the bias (i.e. a reagent change). There is currently a paucity of tools to help investigators effectively and systematically perform quality assessment on large and complex flow cyotmetry data sets [16-18].

### Existing Tools

**BioConductor** provides a suite of open-source tools and software infrastructure to analyze FCM and other high–throughput data [18,19]. The core of this tool set includes **flowCore**, **flowViz**, **flowQ**, and **flowStats**, which together provide functionality for basic data manipulation, visualization, automated gating, and some basic quality control [18,20,21]. The **flowQ** package provides high–level quality control procedures for ungated FCM data using statistical approaches to detect disturbances or unusual patterns in the signals of each channel during acquisition [9]. However, the package is restricted to global measures of quality, as it can only handle ungated data and cannot leverage the complex metadata associated with the larger structure of an FCM study (*e.g.* monitoring the stability of common fluorescence markers in different staining panels of a longitudinal study, or monitoring the variability of gated cell populations across aliquots of a sample).

In order to perform quality assessment of manually gated data, the manual gates and gated data must be accessible to the computational framework for quality assessment. One of the most popular software packages for performing manual FCM gating is **FlowJo** (Tree Star Inc, Ashland, OR). This tool generates “workspace” files in XML format that define the preprocessing and gating applied to a set of FCS files. Currently, the **flowFlowJo** package provides some limited support for importing manually gated data into R from workspace files generated by older Windows–only versions of the software (i.e. FlowJo for Windows version 7.x). However, it does not support workspaces generated by newer versions of **FlowJo** (> version 7), or workspaces generated by **FlowJo** for Mac OS X. Importantly, **flowFlowJo** does not correctly handle **FlowJo**’s specific biexponential data transformation and it is limited to manipulating small data sets that can fit in the available physical memory of the computer [22]. Thus large, real–world FCM data sets generated in clinical studies and data sets analyzed using recent versions of the **FlowJo** or other manual gating tools remain inaccessible to users of BioConductor’s flow tools.

To address these issues, we have developed two new BioConductor packages: **flowWorkspace** and **QUAliFiER** (QUality Assessent for Flow ExpeRiments). **flowWorkspace** makes manually gated data from large, arbitrarily complex FCM studies accessible in the R environment. It imports compensation matrices, data transformations, manual gates, and FCS files from analyses described in **FlowJo** workspaces (supports workspaces generated by **FlowJo** for Mac OS X and Windows), and reproduces them using the BioConductor flow toolset, thus making manually gated data accessible to the computational flow community. The tool has methods implemented for visualizing, summarizing, extracting and exporting population statistics for gated cell populations. Importantly, the tool can handle large FCM data sets through support of NetCDF via the **ncdfFlow** package [23,24]. **flowWorkspace** can also be used to export data to the LabKey (Seattle, WA) tool, allowing one to use R as the engine for flow data analysis with a LabKey front end and data repository [16,17]. The package is closely integrated with other BioConductor flow tools, including normalization via the **flowStats** package and quality control using **QUAliFiER**[20]. **flowWorkspace** makes manually gated flow cytometry data of arbitrarily large size (provided enough disk space is available) accessible for analysis using BioConductor’s flow tools, so that new or automated data analysis strategies can be rapidly compared against current best–practices manual gating methods.

The **QUAliFiER** package uses **flowWorkspace** to import the manual gates defined in the **FlowJo** workspace and calculates summary statistics from each gated cell population. The tool also takes advantage of study metadata describing different samples, aliquots, staining panels and other experimental information to identify outlier samples and cell populations with respect to user–defined grouping variables. For example, the tool can be used to detect instrument variability or changes in reagent quality by monitoring the stability of fluorescence intensities across all samples over the time span of an entire study. These can be monitored in individual channels or in specific cell subpopulations. Another example is use of **QUAliFiER** to assess the consistency of gating specific cell populations in all samples in a study by monitoring the consistency of population statistics for samples derived from a common aliquot and performing outlier detection on cell population frequencies grouped by sample, conditional on the aliquot study metadata. The examples mentioned above are not exhaustive, and QUAliFiER is a general, flexible framework for performing quality assessment flow data that integrates gating information with study–level metadata for each sample. A comparison of the Quality Assurance features available in **flowQ**, **FlowJo**, and **QUAliFiER** is shown in Table 1.

**Table 1:** Comparison of Quality Assurance Functions Available in FlowJo, flowQ, and QUAliFiER

	**Software package**		
**Feature**	**FlowJo**^**1†**^	**flowQ**	**QUAliFiER@**
QA Across Multiple Experiments			*✓*@
QA Ungated Data		*✓*	*✓*@
QA Gated Data			*✓*@
Interactive HTML QA Report		*✓*	*✓*@
Use Study Metadata as Grouping Variables for QA			*✓*@
Customizable QA tasks			*✓*@
Customizable outlier detection			*✓*@
Automated for pipelined analysis		*✓*	*✓*@

^†^Although **FlowJo** does not have internal QA tools, it can export arbitrary population statistics in csv format that may be analyzed in other software.

### Implementation

#### Definitions

In the remainder of the paper we use **bold face** type to refer to **software packages** and teletype font to refer to object, classes, and functions in the packages.

#### Integration

Both packages make use of R’s S4 programming system to define classes and methods, adopting a formal object-oriented paradigm in their implementations [25]. The packages are integrated with the larger flow cytometry package infrastructure available through **BioConductor**. **flowWorkspace** is integrated with the **BioConductor** core flow packages, including **flowCore** for support of the full range of operations on flow data including compensation, transformation, and gating, large data set support through **ncdfFlow** and visualization and plotting through **flowViz**. The **QUAliFiER** package takes advantage of manual gates available through **flowWorkspace** to perform quality assessment of both the gated and ungated FCM data, and produces visualizations of samples flagged as outliers for the further investigation through the **flowViz** package.

#### flowWorkspace

**flowWorkspace** makes use of R’s **XML** package and the *xpath* query language to parse and import **FlowJo** XML workspaces (FlowJo for Mac OS X, versions 7.0 and greater^1^) [26,27]. The package reads in the list of samples, data transformations, compensation matrices, and gates associated with each sample in a workspace and constructs associated **flowCore** objects. The package implements two new data structures to represent this information: the GatingHierarchy and the GatingSet. As the name implies, the GatingHierarchy represents the set of hierarchical gates applied to an individual sample. The GatingSet represents a collection of gated samples from the workspace, analogous to grouped samples in **FlowJo**^2^. However, the design is sufficiently flexible to represent manually gated data coming from any external tool. Each GatingHierarchy is formally a tree data structure associated with a single FCS file, a set of data transformations applied to the channels of the FCS file (these can differ between samples), a compensation matrix (another flow cytometry specific transformation), and a set of gates (boundaries defining distinct cell populations). Each node of the GatingHierarchy tree represents a cell subpopulation in the sample associated with a **flowCore** gate stored at that node. To save space, **flowWorkspace** stores only one copy of the data together with a bit mask representing the events in the sample that are included in each gate.

The data import and gating steps are logically separated, allowing the user to import the workspace without necessarily performing the gating of the data. The package implements parallel import of workspaces using the **parallel** R package, and parallel gating over samples in a workspace using MPI (message passing interface) functionality from the **Rmpi** package [28-30]. The package is available through **BioConductor**http://www.bioconductor.org/packages/2.10/bioc/html/flowWorkspace.html.

#### QUAliFiER

The **QUAliFiER** tool makes use of a local database to store and access extracted cell population statistics from multiple experimental runs (*i.e.* imported **FlowJo** workspaces), as well as study metadata, and resulting outlier calls, allowing QA tasks to span multiple experiments performed in the course of a larger study. The getQAStats function extracts cell population statistics from each sample and gated population defined in a GatingSet and stores them in the local database. The basic quality assessment functionality is defined by the qaTask class, which is a general container that regroups all the information essential to perform a particular quality assessment task. The user pre–defines qaTasks via an external configuration file or directly in an R script that runs the quality assessment procedure, and these are evaluated using the core qaCheck method. This method performs the actual quality assessment for each QA task. Methods for outlier detection, and the specific details of each quality assessment task are all contained in the qaTask object and can be defined by the user or can use any of the pre–defined outlier detection functions or qaTask objects. To visualize the quality assessment results, several plot methods have been implemented including methods for generating dot plots or density plots of gates across samples, and scatterplots or boxplots of population statistics grouped by user–defined or experimental metadata grouping variables. Finally, qa.report collates the generated qaTasks and generates HTML reports with interactive SVG (support vector graphics) graphics and plots for all quality assessment tasks. The package is implemented entirely in *R*, is publicly available on Github as well as **BioConductor**. ( http://mikejiang.github.com/QUALIFIER/, http://bioconductor.org/packages/2.10/bioc/html/QUALIFIER.html).

## Results and Discussion

### 

#### Dataset Description

We present an application of our pipeline to a subset (3000 FCS files) of a large study from the ITN (Immune Tolerance Network) monitoring immunosuppression withdrawal in paediatric recipients of living donor liver transplants [31].

### The QUAliFiER Workflow

The workflow involved in using **QUAliFiER** is relatively straightforward. It involves importing the data, extracting cell population statistics, defining QA tasks, performing outlier calling, and then generating an quality assessment report. The first three steps are handled by the QUALIFIER function, which essentially combines the different pieces of information necessary to perform QA on a dataset. A more detailed description of these steps follows.

#### Importing the QA gating template with flowWorkspace

The code for running the following and other examples can be found in the package source at http://mikejiang.github.com/QUALIFIER.

**Figure 1:**
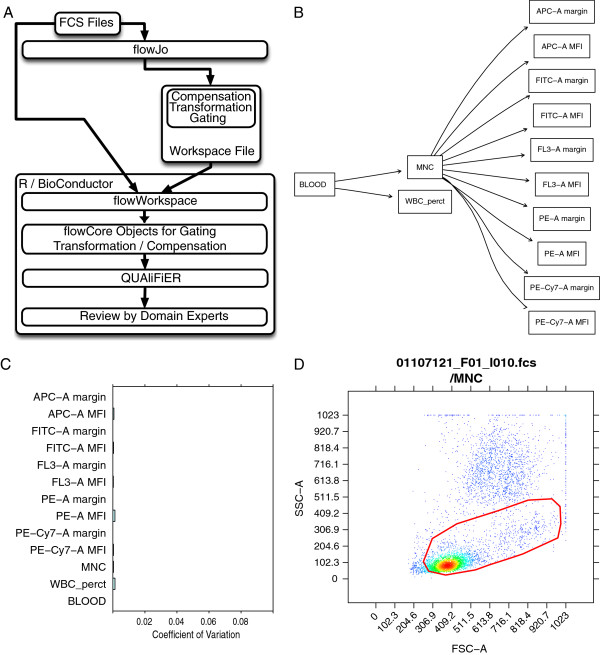
**Functionality of flowWorkspace applied to the QA template gating hierarchy of the test data in the QUAliFiER pipeline.** Functionality of flowWorkspace applied to the QA template gating hierarchy of the test data in the **QUAliFiER** pipeline. **A**) The design of flowWorkspace and its interface with **QUAliFiER**, **FlowJo** and **BioConductor**. **B**) The gating hierarchy for the first sample in the test workspace imported by **flowWorkspace** and displayed via plot. The names of the QA gates defined in the **FlowJo** workspace for the gating template are displayed. This gating template was designed for performing quality assessment of flow data from the ITN (Immune Tolerance Network). MNC is a mononuclear cell gate. WBC_perct is a white blood cell gate. Gates on specific channels with MFI and margin are for detecting the positive populations and boundary events, respectively in each channel. **C**) Agreement between imported population statistics computed using **flowWorkspace** and statistics computed from **FlowJo**, measured via the coefficient of variation between the two values. Slight deviations are due to FlowJo’s discretization of the data transformation function, which must be interpolated by flowWorkspace. Overall the CV is fractions of a percent, indicating successful import. **D**) Example of the dot plots generated by flowWorkspace to visualize gated populations.

The **flowWorkspace** package is used to import the gating template from the **FlowJo** workspace for the ITN study dataset into R. Note that although the workflow presented here uses template gates, the approach can be applied without loss of generality to any set of samples that has been gated in the same manner (i.e. where corresponding populations can be identified across samples). This template includes the set of hierarchical manual gates that identify all the cell sub-poplulations of interest for QA (Figure 1A). A call to openWorkspace creates a flowJoWorkspace object from the XML workspace file, then parseWorkspace reads the template and constructs the necessary R–level objects for the gates, compensation matrices, and data transformations, and optionally reads in the FCS files and performs the preprocessing and gating while calculating the population statistics for each gated cell population. The results for each FCS file are stored in an object of the GatingHierarchy class, with multiple files regrouped in a GatingSet object. This is the object which is ultimately passed on to the **QUAliFiER** package.

The gating hierarchy for any sample can be inspected via plot (Figure 1B) and the success of the import procedure verified via the concordance of the imported cell counts against FlowJo’s cell counts using plotPopCV (Figure 1C). Slight discrepancies (a few fractions of a percent in the coefficient of variation) are due to FlowJo’s quantization of the data transformation function, which must be interpolated by **flowWorkspace**. Larger CVs may either indicate errors in the import process or small (containing few cells) populations where differences of two or three cells between the computed and imported counts result in a large coefficient of variation. Individual gates and samples can be visualized with the plotGate function (Figure 1D) to inspect populations flagged with a large coefficient of variation. Importantly, these statistics and plots can be exported (via ExportTSVAnalysis) to the LabKey tool, which provides a web–based front–end for visualizing gated flow cytometry data [16,17].

#### Extracting population statistics

After importing the data from **FlowJo**, **QUAliFiER** extracts population statistics from the GatingSet (internally via the getQAStats function), and stores them in a local database. Subsequent quality assessment makes use of this database to rapidly query and manipulate the data. **QUAliFiER** can apply filters to the population statistics and perform outlier calls based on grouping and conditioning variables defined in the associated study metadata. Each quality assessment task is defined in a qaTask object. The details for all the qaTasks are provided in a qaTask definition file (described below), whereas the study metadata is supplied as an associated comma separated value file. This file associates each FCS data file with study metadata (e.g. subject, date, dose, aliquot, and so forth). The GatingSet, qaTask definition file, study metadata file, and database connection are passed to the qaPreprocess() function, which does the work of extracting and combining the relevant information from each source into a coherent data structure. Importantly, the **QUAliFiER** package could be used to QA any manually gated data file format supported by **flowWorkspace** and is not limited to the template gating QA process highlighted here. Additionally, **QUAliFiER** could be used in a stand-alone fashion to perform QA on a set of extracted cell-population statistics and study metadata. **flowWorkspace** acts to simplify access to extracted statistics, but is not strictly required for use with **QUAliFiER**.

#### Defining qaTasks

A qaTask defines a specific quality assessment procedure and requires the following information: 

1. The specific cell population or gate for QA.

2. The cell population statistic (i.e. counts or proportion) to QA.

3. The metadata variables for stratification and outlier calling.

4. How to present the data to the user (i.e. plot type).

The qaTask class is a general container that allow users to define different *quality assessment* tasks using the information above. The class uses R’s familiar *formula* interface as a compact and flexible description of the QA task. Briefly, it is generally of the form $y∼x|g_{1}*g_{2}*\ldots \;$, where *y* is the population statistic to monitor and takes four possible values: 

• *MFI*: Median or Mean Fluorescence Intensity of the cell population (the mean or median is user–defined).

• *proportion*: The percent of the parent population represented by the population being QC’d.

• *count*: The number of events in the cell population.

• *spike*: Applicable to each channel of an FCS file measured over the acquisition time. A windowed, cumulative Z–score that quantifies spikes in the MFI of a channel over the acquisition time of the sample. In the absence of spikes, this is approximately zero.

In the right hand side of the formula, *x* specifies the x–axis variable for plotting. It can be any variable defined in the associated study metadata such as date or sample id. Variables on the right of the vertical bar represent *conditioning* variables used to stratify the population statistics for outlier detection. These also must appear in the study metadata. Outlier detection is performed within each level of the cross product of the grouping variables. If these are omitted, then outlier detection is performed on the entire set of samples.

**Figure 2:**
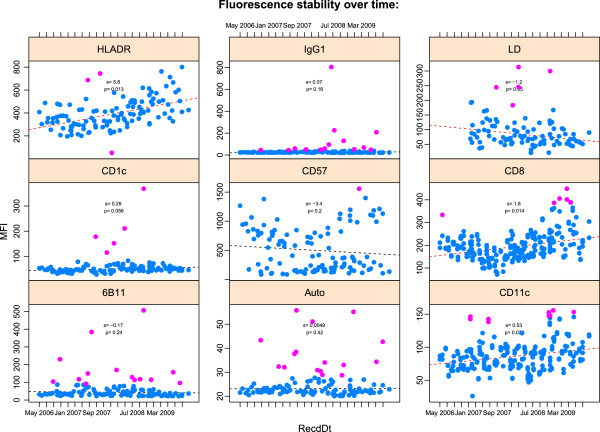
**QA result of of MFI stability vs time for the FITC channel.** We can see clear examples where the MFI is not stable over time, i.e. it is either increasing (HLADR, CD8, CD11c), or decreasing (LD). Some stains show residuals that are not normally distributed, suggesting non-linear trends (HLADR, CD8, CD57), while others are generally stable with the occasional sample outlier (CD1c, IgG1, 6B11). The formula used to generate the plot is: *MFI*∼*RecdDt*|*channel*∗*stain*, where the MFI is plotted against RecdDt, the date the sample was run, which is defined in the study metadata. Channel and stain are generated upon parsing the workspace. Outlier calls are done within each combination of channel and stain. An additional argument to plot(..,subset=channel='FITC',..) tells the function to plot only the output for the *FITC* channel. The population for this qaTask is the population, *MFI*, which selects all MFI gates.

**Figure 3:**
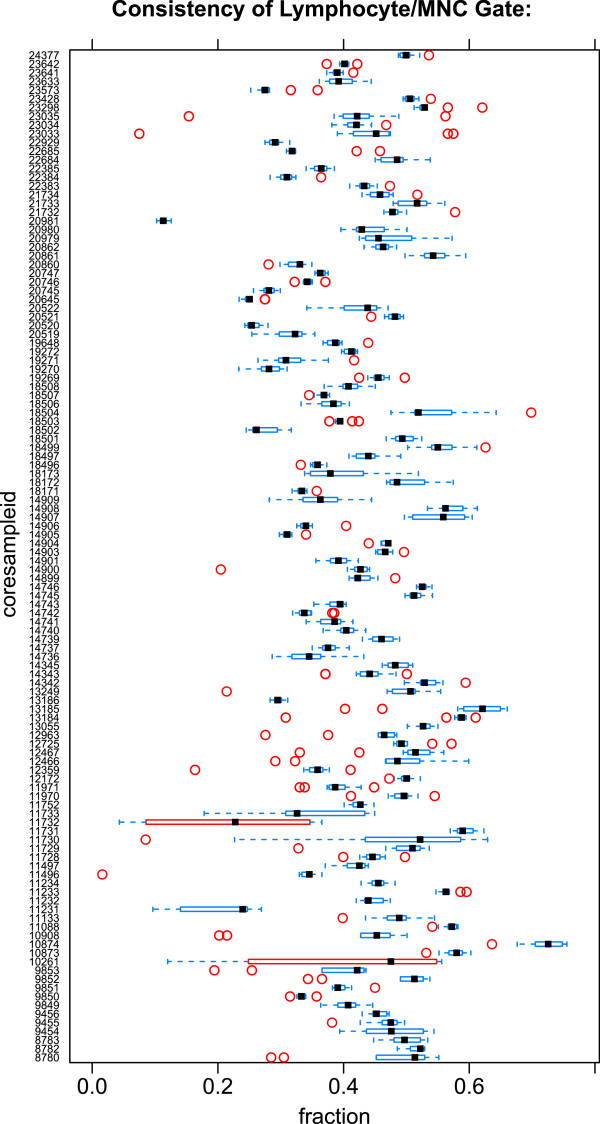
**Consistency of the mononuclear cell gate across aliquots.** The plot shows the consistency of the MNC population across aliquots (*coresampleid*). The plot type is *bwplot*, and the formula for generating this output is *coresampleid*∼*proportion*, while the qaCheck was generated with *proportion*∼*coresampleid*. Additional lattice plot arguments to generate a vertical boxplot layout are passed through the @par slot of the qaTask object. Note that there is no stratification variable. The *bwplot* plot type implies a grouping using the *coresampleid* variable (defined in the study metadata). Boxplots are generated for each level of *coresampleid* and outlier calls (red boxes and points) are made within each group as well as between groups (i.e., identifying groups with larger than expected variability). The population for this qaTask is *MNC*, the mononuclear cell gate.

The qaTask also requires a *plot type* to be specified. This can be any of the standard lattice plot types, such as xyplot or bwplot. The plot type defines how the data will be summarized and presented to the user. **QUAliFiER** defines some default qaTasks such as monitoring the stability of the MFI for a channel over time, or monitoring the variation in the percentage of a cell population within and across aliquots (Figures 2 and 3).

The cell population to be monitored by the qaTask is passed as a name to the pop argument of the qaTask constructor. All of this information (the formula, population name, plot type, and other details) can be provided for all the qaTasks to be performed on a data set via an external csv file passed to the qaPreprocess function. Internally, the makeQaTask function can read a set of these task definitions from the csv file and construct all the qaTask objects simultaneously. Users may also create individual qaTasks directly via the new method.

#### Aggregate QA populations

The population name defining a qaTask generally refers to a unique gated cell population, either via the terminal gate name (e.g. “WBC_perct”), or via a unique gating path (e.g. “/MNC/FITC-A MFI”) (Figure 1B). **QUAliFiER** also supports aggregating populations using common portions of gate names (e.g. “MFI” or “margin”) (Figure 1B). The tool supports regular expression and substring matching to select multiple, non–unique cell populations for QA assessment. In this way, the population “MFI” selects all five terminal populations matching the string “MFI”, which can then be visualized simultaneously in separate plot panels, with each panel representing a different *channel*, as defined in the formula (see Figure 2). Aggregating multiple cell populations in this way for quality assessment provides further flexibility to tailor the quality checks to the needs of the user. This aggregate approach is used throughout the template gates applied to the sample data set in this paper.

#### Outlier Detection and Visualization

Once data is imported and quality assessment tasks are defined, the qaCheck and plot methods perform the quality assessment and visualization based on the definitions stored in each qaTask object.

The actual outlier calls are performed by the qaCheck method. The method reads the population statistics from the database and performs outlier detection within the groups defined in the *formula*. The qaCheck method can accept a default or user–defined outlier detection function.

The package defines several outlier functions for general use in common QA tasks. These are summarized in Table 2. Briefly, the outlier.cutoff function is used to call outliers based on a threshold value of the statistic. The outlier.norm function is used for outlier detection in most QA tasks. It models the data within each group using a normal distribution with a robust estimate of the mean and variance. Outlier calls are made based on a threshold, *α *(significance level) or a Z–score cutoff, either of which can be provided as an argument to the function, which also allows for one or two–sided tests. If the *plot type* is bwplot (box plot), then outlier.norm is used to call between–group outliers (i.e. boxes with a larger than expected variation) with a default Z–score cutoff of 3, based on the distribution of the log–transformed IQRs (Interquartile ranges) of the groups. If the *plot type* is xyplot, the user can add a regression line to the plot via *rFunc* argument (defaults to *rlm* robust regression). Individual observations are flagged as outliers based on the residuals. qoutlier implements the default box–plot outlier detection algorithm for outlier calls within groups for any observation beyond ± 1.5 × IQR for the group.

**Table 2:** Summary of outlier detection methods in the QUAliFiER package

**Outlier function**	**Type**	**Use case**
outlier.cutoff	threshold	1. % of cells in WBC gate for RBC Lysis
		2. % of total events as boundary events
		3. Minimum total event count
outlier.norm	robust normal	1. Stability of MFI of a population vs time
		2. Consistency of gating (%) of a population
		3. High variability groups when measuring between–group variation (i.e. log(IQR) for boxplots)
		4. Individual outliers from residuals of robust regression (i.e. in xyplot)
qoutlier	1.5 × IQR	1. Outliers within groups for boxplots

Outlier detection methods provided by **QUAliFiER** include fixed threshold cutoffs (outlier.cutoff), outlier calls based on t–statistic or Z–score cutoffs or based on significance levels (*α*) (outlier.norm), or calls based on the interquartile range (IQR) of a set of statistics (qoutlier) like that typically used for highlighting outliers in box–plots. Use cases for each are shown in the table.

The qaCheck method will record the outlier calls in the database. Plots can be generated without outlier detection by simply omitting the call to qaCheck. In some applications it may be desirable to simply examine trends rather than make explicit outlier calls (e.g., for monitoring MFI stability over time, Figure 2).

**Figure 4:**
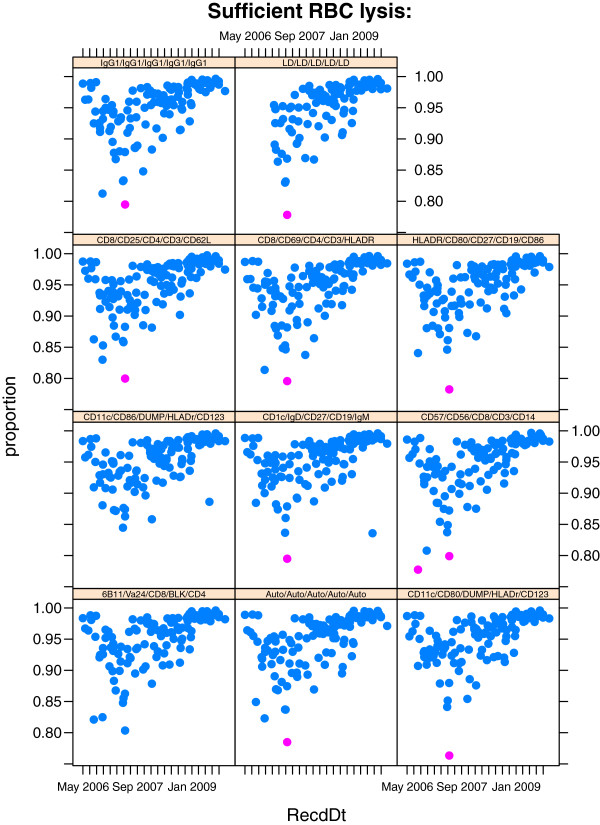
**Consistency of red blood cell lysis across staining panels.** If red blood cells are not properly lysed, they will be detected as events in the FCM experiment. Under ideal conditions, only white blood cells would be detected. The outlier threshold is set such that at least 80% of events should be within the white blood cell gate. Between one and two samples within each staining panel were identified as having lower than expected red blood cell lysis efficiency. Closer inspection revealed these to be from the same *coresampleid*.

We show an example for monitoring the efficiency of red blood cell lysis (Figure 4) from the ITN data set. Efficiency of lysis is measured as the fraction of total cells collected in the WBC_perct (white blood cell) gate (Figure 1B). The qaTask definition used to monitor this population statistic over time, conditioning on all staining panel (tubes) is: 

> data(ITNQASTUDY)

> rbc.lysis<-new(‘‘qaTask'',

pop=‘‘WBC_perct''

,formula=proportion^∼^RecdDt|Tube, qaLevel=‘‘Tube'',

Description=‘‘Sufficient RBC lysis'',

plotType=‘‘xyplot'', qaName=‘‘RBCLysis'', qaID=1L,db=db)

> show(rbc.lysis)

qaTask: RBCLysis

Level : Tube

Description : Sufficient RBC lysis

Plot type: xyplot

Gated node: WBC_perct

Default formula :proportion ^∼^RecdDt | Tube

The call to data loads the study data that has already been parsed and combined with metadata and quality assessment tasks as defined in the previous section. When constructing a qaTask via new it is also necessary to supply a unique qaID, and the database (an environment) holding the extracted statistics and metadata (this is initially passed to the qaPreprocess() function, where it is populated).

To perform the outlier detection, the qaCheck function is called on the *rbc.lysis* task and the results are stored in the database. A call to the plot method will generate the summary plot in Figure 4, passing additional plotting parameters via the par argument. 

> qaCheck(rbc.lysis

,outlierfunc=outlier.cutoff

,lBound=0.8)

> plot(rbc.lysis,

par=list(ylab="percent"))

The plot method is used to generate figures summarizing the outlier detection and quality assessment checks. This function takes the qaTask as an argument, as well as options similar to the **lattice** package, such as subset, which allows a subset of the levels in the grouping variables to be plotted. For example, samples can be subset based on a range of dates, or the plot of the quality assessment task defined above could be restricted to samples within a single staining panel (Tube) by passing subset=Tube%in%'CD8/CD25/CD4/CD3/CD62' to the plot method. This allows for flexibility in visualizing or analyzing subsets of the data.

#### Adding robust regression lines to scatterplots

As data accumulates over the course of a study (e.g. a longitudinal study), **QUAliFiER** stores this data in the QA database, and it becomes trivial to monitor trends in data collected over longer periods of time. As an example, the QA task for monitoring fluorescence stability in the FITC channel over time benefits from the addition of a robust regression line to the output plots within each panel in order to identify groups of samples where there are either non–linear effects or where the MFI is not stable over time (i.e. the slope of the regression is significantly different from zero). The outlier detection task for this procedure is defined in the following way: 

>MFIvsTime<-new("qaTask",

qaName="MFIvsTime",

description=

"Fluorescence stability vs time",

db=db, qaID=2,

qaLevel="Assay",

pop="MFI",

formula=MFI RecdDt|channel⋆stain,

plotType="xyplot")

> qaCheck(MFIvsTime

,outlierfunc=outlier.norm

,rFunc=rlm

,z.cutoff=3)

> plot(MFIvsTime,y=MFI RecdDt|stain

,subset=channel%in%c('FITC-A')

,rFunc=rlm

)

Note the rFunc argument to the qaCheck and plot functions. It allows us to fit a robust linear regression within each group in order to help visualize the changes in MFI over time. Outliers within each level of the *stain* grouping factor are detected based on the deviation of the residuals from the regression line. By default these are called at a threshold of the absolute Z–score of the standardized residuals (3 by default) (Figure 2 and Table 2). If the qaCheck call is omitted, but rFunc is passed to the plot function, the resulting plots will be generated without outlier detection, which may be desirable in some circumstances. Importantly, all the qaTask definitions can be pre–defined in a csv file read in by qaPreprocess(), with column names for each argument to the qaTask constructor.

### Creating a Quality Assessment Report

**Figure 5:**
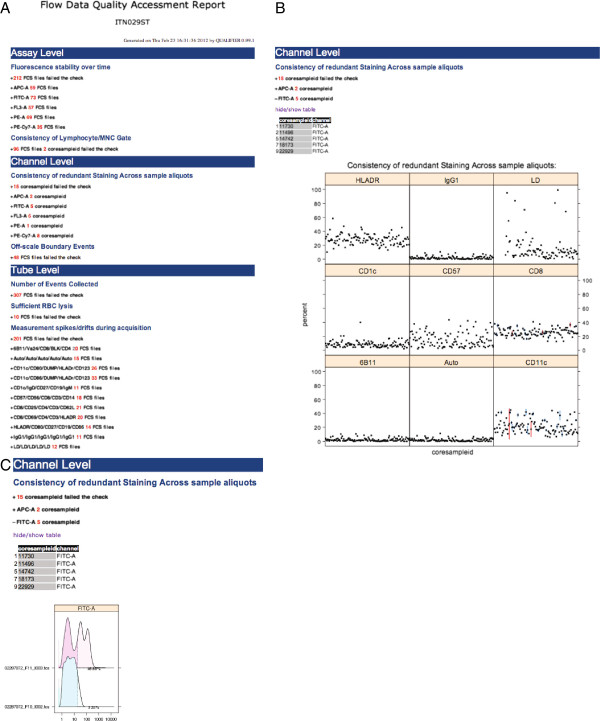
**An xample of the HTML Quality Assessment Report Generated by the QUAliFiER Package.A**) qaTasks are categorized by assay level, channel level, or tube level, depending on the grouping variables for outlier detection. Within each category, a summary of the number of FCS files failing that qaTask is visible. **B**) Clicking on the “+” signs expands a more detailed view of the qaTask, including summary plots and tables. The summary plots themselves are interactive through the use of SVG graphics. The consistency of redundant staining is shown as boxplots of the % of marker positive cells grouped by stain. **C)** Clicking on individual points in the summary plots opens more detailed plots of the cell populations for individual samples failing the qaTask. Densityplots of one of the outlier groups show two samples with inconsistent positive staining in the FITC channel.

The quality assessment tasks for a data set can be summarized via a quality assessment report. This is generated by the qa.report function, which creates an HTML report for all QA tasks defined in a list. The report organizes the results of the qaTasks into categories based on Assay, Tube, Channel, or other user–defined levels (defined by the qaLevel argument of each qaTask). Summary tables of the FCS files failing each qaTask are generated as well as summary plots of each QA task, and low–level, sample specific plots of individual cell populations that failed specific QA tasks (Figure 5). The HTML report makes use of SVG (scalable vector graphics) which allow for user interaction with the data. Generated plots allow mouse–over events to highlight common samples across groups, and include links to more detailed dot plots of specific gates of interest, thus allowing the user to get a global overview of the quality of the data set, and to perform more detailed investigation to identify the root cause of outlier samples. The HTML report provides a convenient interactive mechanism to view and interact with the data in order to get a better understanding of problematic samples or files in the data set. Components of an example report generated from the ITN study data set are shown in Figure 5 and the complete report for the ITN study data set can be found online at http://mikejiang.github.com/QUALIFIER/.

### Summary of the Quality Assessment Report for an ITN Clinical Trial Dataset

**Figure 6:**
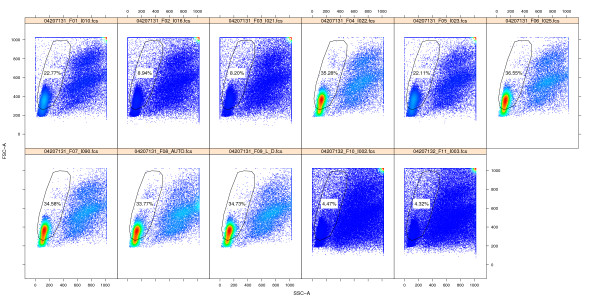
**Dot plots of outlier MNC samples.** Dot plots of the MNC gates for *coresampleid* 11732, one of the group outliers identified by the MNC stability qaTask. Samples with lower proportions of lymphocytes inside the gate are readily visible, caused by elevated debris in the sample.

The **flowWorkspace** and **QUAliFiER** packages were applied to a dataset of 3000 FCS files from the Immune Tolerance Network. The QA report ( http://mikejiang.github.com/QUALIFIER/) identified instances where issues with sample quality merited further review by domain scientists. The stability of the MFI (Figure 2) for each antibody stain showed non–linear effects and changes in stability in some instances, which may have been associated with experimental factors such as changes in the intensity of the staining antibody. The consistency of lymphocyte gating across sample aliquots identified several instances where an elevated amount of debris in the sample resulted in a lower proportion of lymphocytes and mononuclear cells in the MNC gate (Figures 3 and 6). Evaluation of redundant staining (see QA report online, and Figure 5B, C) across sample aliquots allowed for rapid identification of samples with inconsistent staining. The quality of individual aliquots was evaluated by looking at the number of events collected for each aliquot, and identifying those samples where fewer than the number of expected events were collected (see QA report online). Another approach to assess the quality of individual aliquots was to examine the consistency of lysis of red blood cells in each aliquot (Figure 4). Aliquots with fewer than 80% of lysed red blood cells were flagged for further investigation. Instrument stability during sample acquisition was evaluated by monitoring spikes or drifts in each measured channel for each sample (see QA report online). Plots of cumulative Z–scores of those drifts or spikes allowed identification of samples which showed significant deviations.

One of the key advantages of **QUAliFiER** is that it provides an integrated environment for review of quality assurance data by flow domain experts. In the past, the flow analyst would either spot check and manually review plots within flow gating software tools or have data exported from such tools into spreadsheets for sorting, plotting, and viewing of trends over time or across tubes. Should specific anomalies be found, the analyst would have to shuffle between applications, sort through files to review plots within the flow gating tool and return back to summary statistics or plots of trends for confirmation. The disjointed process was cumbersome.

QUALiFiER takes a lot of this frustration out of the process so domain scientists can focus on the scientific questions of interest. It should be noted that the use of QUALiFiER, whether in a research or clinical trial setting is to have the flow cytometry domain expert always review trends and patterns and not simply rely on automatic exclusion of flagged samples. There may be instances where a trend may be due to administration of therapy or other clinical event of interest. In those instances, having the system within the R/BioConducotor framework allows us to easily overlay QA concerns with potentially biological events in an integrated, seamless fashion, further demonstrating the ease and utility of the tool. To our knowledge it is the first tool to integrate this level of extensive quality assessment for large scale gated FCM data in a cohesive pipeline.

Ongoing improvements to the software include complete FlowJo support, as well as FACSDiva (BD Biosciences, San Jose, CA) experiment files, improvements to the HTML report formatting, and generation of PDF output for quality assessment reports. The tool will also be integrated into LabKey (Seattle, WA).

The features and description of the software herein refer to **flowWorkspace** version 1.2 and **QUAliFiER** version 1.0.1 found at the BioConductor website (see Availability and Requirements). The development version of **flowWorkspace** supports Windows and Mac versions of *FlowJo*, including the latest version (version X, Chimera) which is Gating–ML compliant. Support for BD’s (Franklin Lakes, New Jersey) *FACS DiVA* is actively being developed and the next release of *flowWorkspace* will support some the most frequently used manual gating tools (*DiVA* and *FlowJo* reach approximately 50% of users). **FlowWorkspace** data import and gating has also been reimplemented in C++ in the development release, for a 100–fold speed up over the current R–only version of the package.

## Conclusion

**flowWorkspace** is a BioConductor package that allows FCM data that has been preprocessed and manually gated using the *FlowJo* tool (and other tools in future releases) to be imported in the R statistical computing environment where the BioConductor suite of advanced FCM data analysis tools can be leveraged to further analyze the data. A good example of the utility of **flowWorkspace** is its integration with the **QUAliFiER** tool, performing flexible and robust quality assessment of gated and ungated FCM data. Together, the **flowWorkspace** and **QUAliFiER** tools address the increasing demand for tools capable of performing QA on large FCM data sets generated in typical clinical trials. **flowWorkspace** deals with the issue of working with more data than can be loaded into memory at once through its integration with **ncdfFlow** that stores data in netCDF files on disk. **QUAliFiER** provides an infrastructure for identifying outliers amongst the large number of samples collected in an experiment or clinical trial while taking into account the structure imposed by the trial metadata. It simplifies and summarizes the data and presents the results in an interactive way.

The **QUAliFiER** tool automates what has been, for the most part, a manual QA process. Within the ITN, the template gates and subsequent QA are applied manually within flowJo, the resulting statistics extracted, and plots are generated and visualized by an analyst to identify possible problems. In addition, SAS, Excel, and other graphing tools made the process time consuming and disjointed. **QUAliFiER** automates this entire process. The ease of use and customizable nature of the analysis output mark the advantage of the **QUAliFiER** platform over the manual processes. Additionally, **QUAliFiER** brings all the steps of the QA procedure into one software tool. Importantly, **QUAliFiER** is not limited to the template gate-based QA process presented here, but can QA any set of manually gated data (either imported via **flowWorkspace** or otherwise), provided that the data set identifies common cell populations across multiple samples.

**QUAliFiER** objectively, efficiently, and reproducibly identifies outlier samples in an automated manner by monitoring cell population statistics from FCM data conditioned on study–level and experiment–level metadata for outlier detection. The tool has a flexible interface allowing users to define new QA checks and outlier detection routines that suit their data analysis needs. Importantly, interactive quality assessment reports are generated automatically by the tool to facilitate exploration of the data by domain scientists and help identify the underlying causes of potential QA issues flagged by the tool. **QUAliFiER** has uses beyond simple quality assessment. It can be used for exploratory data analysis, to look for correlations between gated populations and clinical covariates for biomarker discovery, and has been applied to evaluate datasets for the *flowCAP* projects ( http://flowcap.flowsite.org/).

## Availability and requirements

**Project name:** QUAliFiER

**Project homepage:**http://mikejiang.github.com/QUALIFIER/

**BioConductor link:**http://bioconductor.org/packages/2.10/bioc/html/QUALIFIER.html

**Operating systems:** Platform independent

**Programming language:** R

**Version:** 1.0.1

**Other requirements:** R, Bioconductor

**License:** Artistic 2.0

**Project name:** flowWorkspace

**Project homepage:**http://github.com/gfinak/flowWorkspace

**BioConductor link:**http://bioconductor.org/packages/2.10/bioc/html/flowWorkspace.html

**Operating systems:** Platform independent

**Programming language:** R and C++

**Version:** 1.2.0

**Other requirements:** R, Bioconductor

**License:** Artistic 2.0

## Abbreviations

FCM: (flow cytometry); SVG: (scalable vector graphics); ITN: (Immune ToleranceNetwork).

## Competing interests

The authors declare that they have no competing interests.

## Author’s contributions

RG and GF and WJ developed the methodology and designed the software. WJ and GF developed the software, and performed the analyses. AA and JP participated in its design and coordination. WJ and GF drafted the manuscript. All authors read and approved the final manuscript.

## References

[B1] Braylan RC Impact of flow cytometry on the diagnosis and characterization of lymphomas, chronic lymphoproliferative disorders and plasma cell neoplasias Cytometry A 2004 58A 57 61 10.1002/cyto.a.10101 14994222

[B2] Hengel RL Nicholson JK An update on the use of flow cytometry in HIV infection and AIDS Clin Lab Med 2001 21 4 841 856 11770291

[B3] Illoh OC Current applications of flow cytometry in the diagnosis of primary immunodeficiency diseases Arch Pathol Lab Med 2004 128 23 31 14692816 10.5858/2004-128-23-CAOFCI

[B4] Kiechle FL Holland-Staley CA Genomics, transcriptomics, proteomics, and numbers Arch Pathol Lab Med 2003 127 9 1089 1097 12946210 10.5858/2003-127-1089-GTPAN

[B5] Mandy FF Twenty-five years of clinical flow cytometry: AIDS, accelerated global instrument distribution Cytometry A 2004 58A 55 56 10.1002/cyto.a.10102 14994221

[B6] Orfao A Ortuno F de Santiago M Lopez A San Miguel J Immunophenotyping of acute leukemias and myelodysplastic syndromes Cytometry A 2004 58A 62 71 10.1002/cyto.a.10104 14994223

[B7] Bagwell CB DNA histogram analysis for node-negative breast cancer Cytometry A 2004 58A 76 78 10.1002/cyto.a.90004 14994225

[B8] Keeney M Gratama JW Sutherland DR Critical role of flow cytometry in evaluating peripheral blood hematopoietic stem cell grafts Cytometry A 2004 58A 72 75 10.1002/cyto.a.10103 14994224

[B9] Bashashati A Brinkman RR A survey of flow cytometry data analysis methods Adv Bioinf 2009 584603 2009 10.1155/2009/584603 PMC2798157 20049163

[B10] Krutzik PO Irish JM Nolan GP Perez OD Analysis of protein phosphorylation and cellular signaling events by flow cytometry: techniques and clinical applications Clin Immunol 2004 110 3 206 221 10.1016/j.clim.2003.11.009 15047199

[B11] Maecker H McCoy P Nussenblatt R Standardizing Immunophenotyping for the Human Immunology Project Nat Rev Immunol 2012 12 3 191 200 22343568 10.1038/nri3158 PMC3409649

[B12] Pozarowski P Darzynkiewicz Z Analysis of cell cycle by flow cytometry Methods Mol Biol 2004 281 301 312 15220539 10.1385/1-59259-811-0:301

[B13] Pala P Hussell T Openshaw PJ Flow cytometric measurement of intracellular cytokines J Immunol Methods 2000 243 1-2 107 124 10.1016/S0022-1759(00)00230-1 10986410

[B14] Vermes I Haanen C Reutelingsperger C Flow cytometry of apoptotic cell death J Immunol Methods 2000 243 1-2 167 190 10.1016/S0022-1759(00)00233-7 10986414

[B15] Lehmann AK Sornes S Halstensen A Phagocytosis: measurement by flow cytometry J Immunol Methods 2000 243 1-2 229 242 10.1016/S0022-1759(00)00237-4 10986417

[B16] Shulman N Bellew M Snelling G Carter D Huang Y Li H Self SG McElrath MJ De Rosa SC Development of an automated analysis system for data from flow cytometric intracellular cytokine staining assays from clinical vaccine trials Cytometry Part A : j Int Soc Anal Cytology 2008 73 9 847 856 10.1002/cyto.a.20600 PMC2591089 18615598

[B17] Nelson EK Piehler B Eckels J Rauch A Bellew M Hussey P Ramsay S Nathe C Lum K Krouse K Stearns D Connolly B Skillman T Igra M LabKey Server: an open source platform for scientific data integration, analysis and collaboration BMC Bioinf 2011 12 71 10.1186/1471-2105-12-71 PMC3062597 21385461

[B18] Hahne F Le Meur N Brinkman R Ellis B Haaland P Sarkar D Spidlen J Strain E Gentleman R flowCore: A Bioconductor software package for high throughput flow cytometry data analysis BMC Bioinf 2009 10 106 10.1186/1471-2105-10-106 PMC2684747 19358741

[B19] Gentleman RC Carey VJ Bates DM Bolstad B Dettling M Dudoit S Ellis B Gautier L Ge Y Gentry J Hornik K Hothorn T Huber W Iacus S Irizarry R Leisch F Li C Maechler M Rossini AJ Sawitzki G Smith C Smyth G Tierney L Yang JYH Zhang J Bioconductor: Open software development for computational biology and bioinformatics Genome Biol 2004 5 10 R80 10.1186/gb-2004-5-10-r80 15461798 PMC545600

[B20] Hahne F Khodabakhshi A Bashashati A Wong CJ Gascoyne RD Weng A Seyfert-Margolis V Bourcier K Asare A Lumley T Gentleman R Brinkman R Per-channel basis normalization methods for flow cytometry data Cytometry Part A 2010 77A 121 131 10.1002/cyto.a.20823 PMC3648208 19899135

[B21] Sarkar D Le Meur N Gentleman R Using flowViz to visualize flow cytometry data Bioinformatics 2008 24 6 878 879 10.1093/bioinformatics/btn021 18245128 PMC2768483

[B22] Gosink JJ Means GD Rees WA Su C Rand HA Bridging the Divide between Manual Gating and Bioinformatics with the Bioconductor Package flowFlowJo Advances in Bioinformatics 2009 809469 2009 10.1155/2009/809469 PMC2775689 19956421

[B23] Rew R Davis G NetCDF: an interface for scientific data access Computer Graphics and Applications, IEEE 1990 10 4 76 82

[B24] Mike Jiang NG Greg Finak ncdfFlow: ncdfFlow: A package that provides ncdf based storage for flow cytometry data [ http://bioconductor.org/packages/2.10/bioc/html/ncdfFlow.html]

[B25] Chambers JM Programming with Data: A Guide to the S Language 2004 175 Fifth Avenue, NY 10010, USA: Springer–Verlag, New York, Inc.

[B26] Lang DT XML: Tools for parsing and generating XML within R and S-Plus 2012, [ http://CRAN.R-project.org/package=XML]. [R package version 3.9-4]

[B27] Chan C Felber P Garofalakis M Efficient filtering of XML documents with XPath expressions VLDB J—Int J Very Large Data Bases 2002 11 4 354 379 10.1007/s00778-002-0077-6

[B28] Yu H Rmpi: Interface (Wrapper) to MPI (Message-Passing Interface) 2010, [ http://CRAN.R-project.org/package=Rmpi]. [R package version 0.5-9]

[B29] Knaus Jsnowfall: Easier cluster computing (based on snow) 2010, [ http://CRAN.R-project.org/package=snowfall]. [R package version 1.84]

[B30] Urbanek Smulticore: Parallel processing of R code on machines with multiple cores or CPUs 2011, [ http://CRAN.R-project.org/package=multicore]. [R package version 0.1-7]

[B31] Feng S Ekong U Lobritto S Clinical and histological predictors of operational tolerance in pediatric liver transplant recipients The 2011 Joint International Congress of ILTS , ELITA , &amp; LICAGE Valencia, June 22-25, 2011; Spain Edited by Lake, J R and Roberts, JP 17 suppl 1 S10721618695

